# Pulmonary midkine inhibition ameliorates sepsis induced lung injury

**DOI:** 10.1186/s12967-021-02755-z

**Published:** 2021-02-27

**Authors:** Jing-Yuan Xu, Wei Chang, Qin Sun, Fei Peng, Yi Yang

**Affiliations:** Jiangsu Provincial Key Laboratory of Critical Care Medicine, Department of Critical Care Medicine, Zhongda Hospital, School of Medicine, Southeast University, 87 Dingjiaqiao Rd., Nanjing, 210009 People’s Republic of China

**Keywords:** Midkine, Angiotensin converting enzyme, Sepsis, Angiotensin II

## Abstract

**Background:**

Midkine is a multi-functional molecule participating in a various key pathological process. We aimed to evaluate the change of midkine in sepsis and its association with angiotensin-converting enzyme (ACE) system, as well as the mechanism by which midkine induced in sepsis and lung injury.

**Methods:**

The peripheral blood sample of septic patients on admission was obtained and measured for midkine, ACE and angiotensin II. Cecal ligation and puncture (CLP) mouse model was used, and adeno-associated virus (AAV) was stilled trans-trachea for regional targeting midkine expression, comparing the severity of lung injury. Furthermore, we studied the in vitro mechanism of midkine activates ACE system by using inhibitors targeting candidate receptors of midkine, and its effects on the vascular endothelial cells.

**Results:**

Plasma midkine was significantly elevated in sepsis, and was closely associated with ACE system. Both circulating and lung midkine was increased in CLP mouse, and was related to severe lung injury. Regional interfering midkine expression in lung tissue by AAV could alleviate acute lung injury in CLP model. In vitro study elucidated that Notch 2 participated in the activation of ACE system and angiotensin II release, induced by midkine and triggered vascular endothelial injury by angiotensin II induced reactive oxygen species production.

**Conclusions:**

Midkine inhibition ameliorates sepsis induced lung injury, which might via ACE/Ang II pathway and the participation of Notch 2 in the stimulation of ACE.

*Trial registration*

Clinicaltrials.gov NCT02605681. Registered 12 November 2015

## Introduction

Sepsis is a great challenge in the intensive care units (ICU) with mortality over 40% once precipitates to multiple organ dysfunction syndrome despite advanced progresses in early resuscitation, antibiotic use and organ protective therapeutic strategies [1, 2], and remains a great burden to the public health [3].

The definition of sepsis involves the dysregulated host immune response to microbial pathogen assaults [4]. Vascular endothelium is one of the primary targets under attack by the abnormally activated immune system [5]. Vascular endothelial cell injury, characterized by glycocalyx shedding [6], endothelium integrity damage [7], and inflammatory adhesive molecule activation [8], presented as increased expression of intercellular junction molecules including vascular endothelial (VE)—cadherin to elucidate the permeability of vascular endothelium, and vascular cell adhesive molecule (VCAM)—1 to clarify the inflammation of endothelial cells, are closely correlated with organ dysfunction and mortality in sepsis [9].

Midkine, a 13-kDa cysteine-rich polypeptide, is a multi-functional factor predominantly secreted in embryogenesis but little expressed in healthy adult organs [10]. Plasma midkine is reported to be elevated in sepsis by Krzystek-Korpacka et al. in a clinical observational study, and is associated with sepsis severity [11]. Our previous work also found associations between plasma midkine and 28-day mortality and organ dysfunction in sepsis [12].

Study has demonstrated that midkine could regulate angiotensin-converting enzyme (ACE) in a murine hypertensive model induced by partial nephrectomy. Genetic ablation of midkine in mouse is proved to suppress ACE mRNA and protein expression, and alleviates hypertension induced kidney injury [13], implying its plausible role in renin-angiotensin system (RAS) system regulation.

Angiotensin-converting enzyme, an enzyme with hydrolytic ability mainly distributed in the pulmonary capillary bed, which converses the substrate angiotensin I to the end-product angiotensin II by cleaving dipeptides of histamine—leucine from the carboxyl terminal of angiotensin I [14]. Angiotensin II has been found profoundly elevated in sepsis [15], which correlated with organ dysfunction and outcomes in septic patients [16].

The detrimental effects to the vascular endothelial cells by angiotensin II have long been established [17]. Angiotensin II injures the endothelial cells by electron transport uncoupling, nicotinamide adenine dinucleotide phosphate (NAPDH) oxidation and reactive oxygen species (ROS) release, which damage the vascular endothelial cells, ultimately leads to organ dysfunction [18]. In our previous work, Liu et al. implys that lung injury severity was associated with ACE activity and angiotensin II level in an experimental lipopolysaccharide (LPS)—induced acute lung injury (ALI) model, but was ameliorated by using losartan, an ACE inhibitor [19].

However, the explicit role of midkine in sepsis and the mechanism by which midkine regulates ACE remains unclear. Some of the candidate receptors have been reported to participate in midkine signaling pathway, including anaplastic lymphoma kinase (ALK), epidermal growth factor receptor (EGFR) and Notch 2 [20–22], which needs further validation.

In this study, we aim to investigate the correlation between midkine and ACE system in septic patients. We subsequently studied the impact of midkine depletion in sepsis induced ALI, using a midkine RNAi adeno-associated virus (AAV) through orotracheal injection in a mouse cecal ligation puncture (CLP) model. We further studied the in vitro mechanism by which midkine caused endothelial injury.

## Methods

### Ethics statement

The study was performed under the approval of the Research Ethic Committee of Zhongda Hospital (Southeast University, Nanjing, China, 2015ZDSYLL069.0), and registered online (NCT02605681). The written consents were signed by the close relatives of the patients, and the healthy volunteers. All animal experiments performed in this study conformed to the Guide for the Care and Use of Laboratory Animals and were approved by the Institutional Animal Care and Use Committee of Southeast University.

### Patients

Patients diagnosed with sepsis according to the Surviving Sepsis Campaign (2016) guidelines [23] were recruited in the prospective cohort study from November 2017 to March 2018, admitted to the Department of Critical Care Medicine, Zhongda Hospital, a tertiary hospital. All patients were followed-up to day-28 and the all-cause mortality was recorded.

The peripheral blood sample were obtained immediately after admission with ethylenediamine tetraacetic acid (EDTA) anti-coagulation tube, centrifuged with the plasma separated and stored at – 80 ℃ for batch analysis. Plasma ACE and angiotensin II were measured by enzyme-linked immunosorbent assays (ELISA) (Elabscience, China) blind to the information of the patients. Plasma midkine (CUSABIO, China) were measure as well. The patients were followed-up to 28 days. Additional 5 healthy volunteers were recruited as health control.

### Mice

Forty-eight C57BL/6 male mice aged 3 to 4-week-old from the Experimental Animal Center (Yangzhou University, Yangzhou, China) were housed and used under sterile conditions at the Research Center of Genetically Modified Mice (Southeast University, Nanjing, China). The mice were routinely fed and maintained at the constant temperature (22 ℃), with 12-h light and dark cycles. The body weights of the mice were randomized and assigned to different treatment groups.

### Adeno-associated virus establishment and injection

The AAV vector (Genechem, China) carrying midkine RNA interfering sequence was established (titters were adjusted to 1–2 × 10^12^ viral genomes per milliliter for injection). The virus was instilled from orotracheal to the 3-to 4-week-old mice for intervention of pulmonary midkine expression, with the volume of 10μL, and the maximum virus load of 2 × 10^10^ viral genome, and equivalent viral loads carrying control sequence was given to the control vector group. The animals were fed ad libitum and maintain at 22 ℃. The efficacy of transduction was validated by immunofluorescence staining and the efficacy of gene interference was determined by RT-qPCR and Western blots, both 4 weeks after injection.

### Cecal ligation and puncture model of sepsis

The mouse CLP model was established as previous described [24]. Briefly, the mouse was anesthetized with ketamine (100 mg per kg body weight) and xylazine (10 mg per kg body weight) by intraperitoneal injection. The lower quadrant of abdomen was shaved and sterilized with 70% ethanol. The mouse was positioned in dorsal recumbency, with head oriented away from the operator. Midkine incision (~ 2 cm) was made through the skin and linea alba into the abdominal cavity. The cecum was located, exteriorized and ligated at ~ 1 cm from the apex. A single through-and-through puncture with a 22-gauge needle was made at the middle between the ligation and the apex, small droplets of the cecal contents were extruded. The cecum was relocated into the abdominal cavity and peritoneum and skin were closed. Sham surgery was performed on the control mouse, with the cecum exteriorized as described above but not ligated or punctured. Prewarmed normal saline (37 ℃, 50 ml/kg) was injected subcutaneously for resuscitation immediately after surgery. Established CLP mice were featured as malaise, piloerection, generalized weakness and reduced gross motor activity. The mouse was fed ad libitum and euthanized 24 h after surgery.

### Histological analysis

Lung was removed from the mouse, fixed in 10% formalin, embedded in paraffin, sectioned, mounted onto the slide and stained with hematoxylin–eosin (H&E). We randomly chose 5 high-power fields (HPFs, × 200 magnification) (Olympus, Japan) per section for each mouse, evaluated by 2 individual qualified pathologists blinded to the mouse grouping. Lung injury was graded and quantified according to the following categories including alveolar congestion, hemorrhage, infiltration of inflammatory cells, and hyaline membrane formation, as previously described [25]. Sections were blocked with 5% bovine serum albumin in phosphate buffer solution (PBS) (Wisent, Canada) for 1 h at room temperature and incubated with primary antibodies GFP (1/500) (CST, USA) or midkine (1/40) (R&D Systems, USA) overnight at 4 ℃. After washing with PBS, sections were incubated in appropriate fluorescent secondary antibodies (1/500) or HRP-conjugated secondary antibodies (1/500) in PBS for 1 h at room temperature.

### Bronchoalveolar lavage fluid analysis

Protein contents and total cell counts were evaluated by bronchoalveolar lavage fluid (BALF) analysis. The mouse was sacrificed by exsanguination, and the trachea was exposed and intubated with 22-gauge catheter, with 3 repeats of injection of total 1 ml PBS, as previously described [26]. The BALF was recovered and centrifugated (500 g, 4 ℃, 20 min); the supernatant was collected with further centrifugation (16500 g, 10 min, 4 ℃) and stored at −80 ℃ for batch analysis of protein contents by bicinchoninic acid (BCA) methods (Beyotime, China); while the cells precipitated in the pellet was resuspended in PBS for subsequent total cell count by the handled automated cell counter (Millipore, USA). Tumor necrotic factor-α (TNF-α) was measured in the BALF by immunoassays (Elabscience, China) following the manufacturer’s protocols.

### Lung wet/dry ratio

Lung samples were dissected and weighted immediately. The samples were desiccated at 60 ℃ for 48 h till reaching a constant weight. The ratio of wet to dry ratio of the lung was calculated to determine the pulmonary edema [25].

### Myeloperoxidase activity

The pulmonary neutrophil infiltration was determined by Myeloperoxidase (MPO) activity in the homogenized lung tissue with MPO Detection Kit (Jiancheng Bioengineering, China). Lung tissue was homogenized in 50 nM potassium phosphate buffer saline (pH 6.0) containing 0.5% hexadecyltrimethylammonium hydroxide and transferred into PBS (pH 6.0) containing 0.17 mg/ml 3,3′-dimethoxybenzidine and 0.0005% hydrogen peroxide solution. MPO activity was determined by measuring the H_2_O_2_-dependent oxidation of 3,3′-dimethoxybenzidine and expressed as units per gram of wet tissue (U/g) [27].

### Cell

Pulmonary microvascular endothelial cell (PMVEC) (Procell, China) was cultivated in the endothelial cell medium (ECM) (ScienCell, USA) containing 10% fetal bovine serum (FBS) (Wisent, Canada), endothelial cell growth supplements (ECGS) (ScienCell, USA) and 1 mM penicillin/streptomycin solution (Wisent, Canada). The cells were starved overnight at 60–70% confluence. The cells were treated with recombinant midkine (100 ng/ml in water, PeproTech) for 36 h, and subsequently incubated with angiotensin I (1 mM in dimethyl sulfoxide, DMSO) (Tocris bioscience, UK) for 24 h.

### Reagents

Cells were pre-treated with inhibitors including DAPT (Notch inhibitor, 100 nM in DMSO) (AbCam, USA), Erlotinib (EGFR inhibitor, 2 nM in DMSO) (Selleck, USA) or LDK378 (ALK inhibitor, 0.2 nM in DMSO) (MCE, USA) for 12 h before stimulated by midkine. Enalaprilat (2 nM in DMSO) (Selleck, USA) was used as ACE inhibitor.

### RNA extraction and real time—quantitative PCR

Total RNA was isolated from the tissue homogenization or cells according the manufacturer’s protocols. The total RNA was quantified and 1 μg of RNA as reversely transcribed by HiScript II Q RT SuperMix reverse transcription kit (Vazyme Biotech, China). Following sequence was synthesized as primers, forward, 5′-CAAGGGACCCTGAAGAAGGC-3′, and reverse, 5′-CTTTGGTCTTTGACTTGCTCTTGG-3′ for midkine, and forward, 5′-ACCCAACCTCGATGTCACCA-3′, and reverse, 5′-GCGAGGTGAAGAATTCCTCTGA-3′ for ACE. Reactions were performed with a 20μL SYBR GREEN PCR volume by AceQ Universal SYBR qPCR kit (Vazyme Biotech, China) in ABI OneStep (Applied Biosystems), with beta-actin as the internal control for RNA differences among each sample.

### Western blotting

The total protein in the tissue homogenization or cells lysate was quantified by BCA and underwent SDS-PAGE (Beyotime, China) and electro-transferred to PVDF membranes (Millipore, USA). After being blocked by 5% BSA TBS-T solution, membranes were incubated with primary antibodies against midkine (1/200) (R&D Systems, USA), ACE (1/500) (AbCam, USA), VE-Cadherin (1/1000) (CST, USA) and VCAM-1 (1/1000) (AbCam, USA), respectively. The protein was visualized by chemiluminescence with ECL kit (Beyotime, China), and quantified by densitometry, which were normalized to house-keeping protein of β-actin.

### Angiotensin converting enzyme activity

Angiotensin converting enzyme activity was evaluated by N-[3-(2-furyl) acryloyl]-L-phenylalanyl-glycyl-glycine (FAPGG, 2 mM, dissolved in Tris phosphate buffer, pH 8.0, [Cl^−^] was tittered to 600 mM using 10% sodium chloride solution) (Sigma Aldrich, USA). The treated cells or lung homogenization were planted in the 96-well plate of 100 μl, added with 100 μl of FAPGG solution, and incubated at 37 ℃. The optical absorbance at 340 nm was measured at 5 min intervals from time 0 till 1 h. The absolute value of the optical absorbance at 340 nm of the subtract at the indicated time point to time zero was calculated to determine ACE hydrolytic activity.

### Cell viability

Cells were treated with the fresh culture medium with angiotensin I (R&D Systems, USA). Cell viability was assessed by a Cell Counting Kit-8 assay (Beyotime, China), according to manufacturer’s instructions. Briefly, after treatment, the CCK-8 solution was added to the culture medium and incubated for 1 h. The absorbance was read at 450 nm with a microplate reader (Thermo Fisher, USA). Cell viability was calculated by experimental group absorbance value/control group absorbance value.

### Immunofluorescence

Pulmonary microvascular endothelial cells were seeded on the upper chambers of 0.4-μm cell-culture inserts and cultured for 1–3 days to allow the growth of a confluent monolayer. Then cells were washed in cold phosphate-buffered saline (PBS) and fixed in 4% paraformaldehyde. Non-specific binding of antibody was blocked in 1% BSA in PBS for 1 h. Cells were stained with vascular endothelial VE-cadherin antibody (1:500) (Santa Cruz, USA), ZO-1 antibody (1:200) (R&D Systems, USA), VCAM-1 antibody (1:500) (Santa Cruz, USA) and then washed with PBS. DAPI (4,6- diamidino-2-phenylindole) (Sigma-Aldrich, USA) was used to stain nuclei. Samples were examined with a microscope (Olympus, Japan) equipped with fluorescent illumination.

### Statistics

Continuous variable with normal distribution was presented as mean ± standard deviation (SD) or standard error (SE), while skewed distribution was presented with median (interquartile range, IQR). Continuous variables were compared by student* t* test or Mann–Whitney *U* test between two groups and one-way analysis of variance (ANOVA) or Kruskal–Wallis test within three or more groups. Pairwise comparisons were made by post hoc analysis. Binary variable was presented as frequencies and compared by chi-square test or Fisher’s exact test. The data were computerized and analyzed by Prism GraphPad and IBM SPSS Statistic Package. A 2-tailed threshold of 0.05 was considered as statistical significance.

## Results

### Plasma midkine was elevated in patients with sepsis

**Fig. 1:**
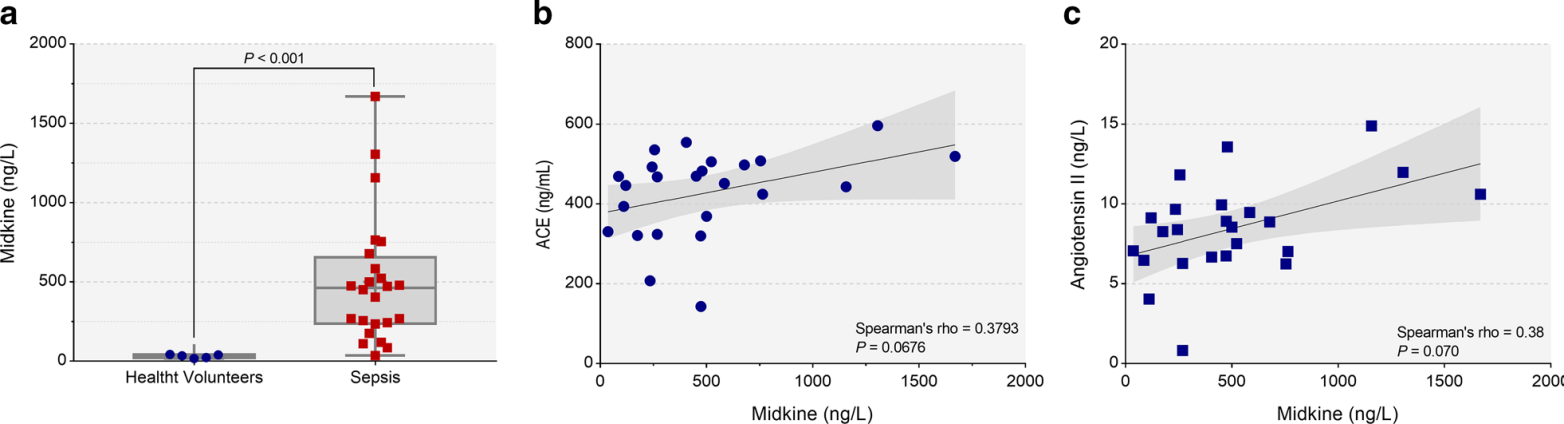
Clinical Profiles. **a** Plasma midkine remarkedly elevated in septic patients (*n* = 26) vs. healthy volunteers (*n* = 5). **b** The correlation between plasma midkine and ACE (Spearmen’s *rho* = 0.3793, *P* = 0.0676). **c** The correlation between plasma midkine and angiotensin II (Spearmen’s *rho* = 0.38, *P* = 0.07)

A total of 26 septic patients were finally enrolled in the study, with 18 survivors and 8 non-survivors followed-up to day-28 (detailed flowchart, see Additional file 1: Fig. S1). Additional 5 healthy volunteers were also recruited. Baseline characteristics were shown in Table 1. Plasma midkine was strikingly elevated in septic patients compared with healthy volunteers [461.9 (236.8–654.9) vs. 34.3 (20.7–41.2), *P* < 0.001] (Fig. 1a). No significant correlations could be observed between plasma midkine and ACE or angiotensin II in septic patients, with *P*-values for linear correlation of 0.0676 (Fig. 1b) and 0.07 (Fig. 1c), respectively.

**Table 1:** Baseline characteristics

	Septic patients (n = 26)	Healthy volunteers (n = 5)
Age, mean ± SD, year	63 ± 19	45 ± 8
Male, n (%)	21 (81)	4 (80)
SOFA score, mean ± SD	8.7 ± 3.8	-
Infection sources		
Pulmonary, n (%)	15 (57.7)	-
Abdominal, n (%)	7 (26.9)	-
Other, n (%)	4 (15.4)	-
Septic shock, n (%)	19 (73.1)	-
Vasopressor, n (%)	20 (76.9)	-
Mechanical ventilation, n (%)	20 (76.9)	-
CRRT, n (%)	7 (26.9)	-
28-day mortality, n (%)	8 (30.8)	-

*SOFA* sequential organ failure assessment, *CRRT* continuous renal replacement therapy, *SD* standard deviation

*p < 0.05

### Midkine expression was elevated in CLP mouse

**Fig. 2:**
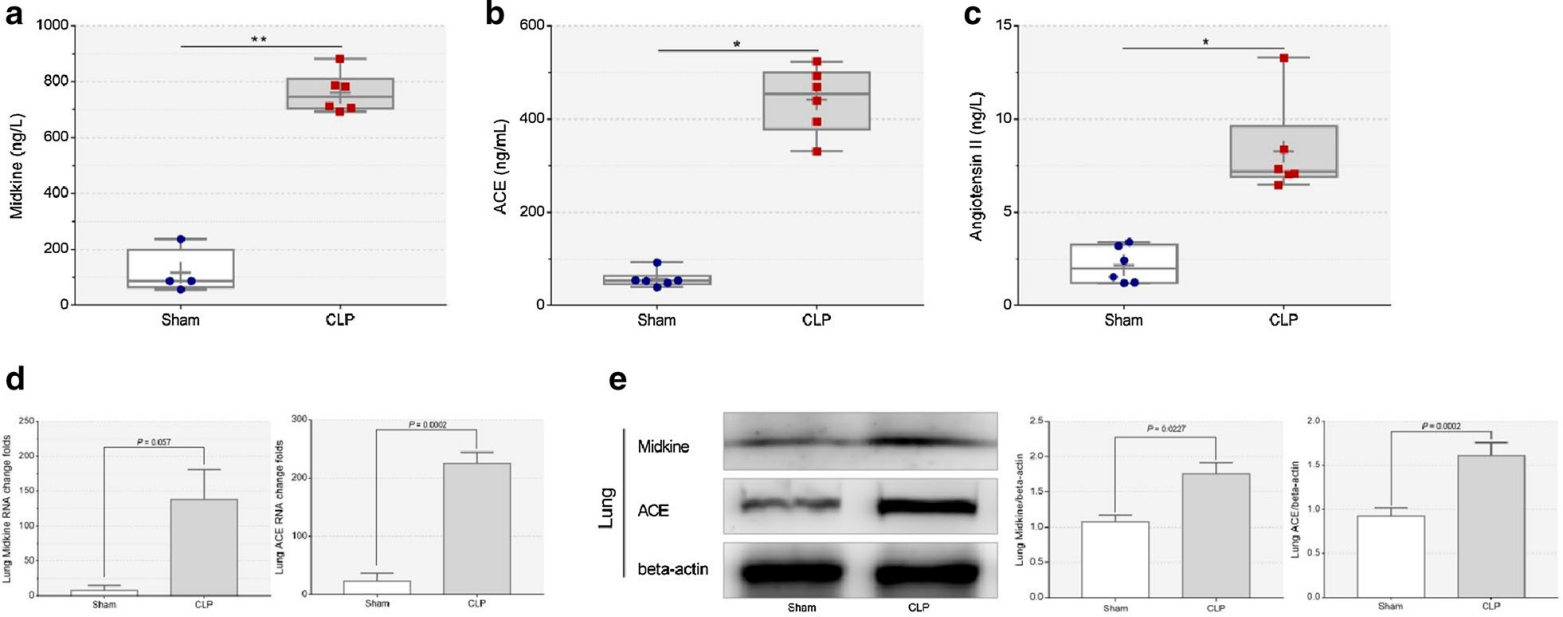
Midkine and ACE level in plasma and lung tissue. **a** Plasma midkine in CLP (*n* = 6) vs. sham (*n* = 6) model [ng/L, 747.3 (703.6–811.1) vs. 87 (64.5–199.5), *P* = 0.0095]. **b** Plasma ACE in CLP (*n* = 6) vs. sham (*n* = 6) [ng/mL, 454.1 (378.1–500.3) vs. 53.4 (46.3–63.8), *P* = 0.0022]. **c** Plasma angiotensin II in CLP (*n* = 6) vs. sham (*n* = 6) [ng/L, 7.2 (6.8–9.6) vs. 1.9 (1.2–3.3), *P* = 0.002]. **d** Midkine and ACE mRNA level in lung tissue in CLP (*n* = 6) vs. sham (*n* = 6), mRNA amplification was normalized to β-actin. **e** Midkine and ACE protein level was determined by Western blots, and the representative result was shown. Quantitative analysis of midkine and ACE protein using densitometry, normalized to β-actin

Plasma midkine was markedly elevated in CLP mouse (*n* = 6) compared with sham (*n* = 6) [ng/L, 747.3 (703.6–811.1) vs. 87 (64.5–199.5), *P* = 0.0095, Fig. 2a]. Midkine expression in lung tissue was induced in CLP mouse (Fig. 2d, e). All the collective results suggested that midkine in plasma and lung tissue was significantly increased in the CLP model.

**Fig. 3:**
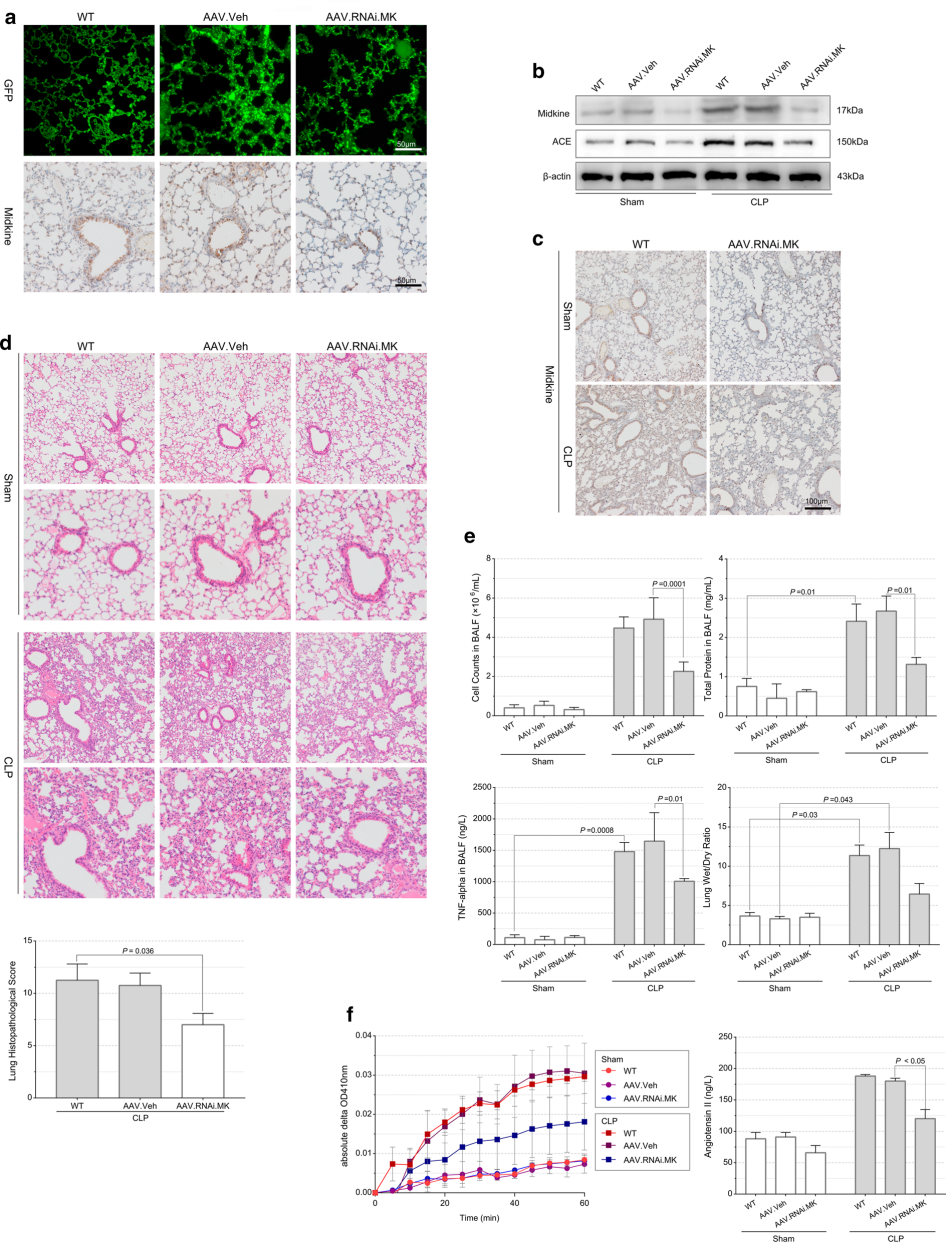
Targeted abrogation of midkine in the lung tissue ameliorated acute lung injury in CLP model. **a** Immunofluorescence staining of GFP of lung tissue in control mouse (WT), mouse transduced with vehicle AAV (AAV. Veh) and mouse transduced with AAV carrying midkine RNAi sequence (AAV.RNAi.MK, *n* = 6 in each group). Lower panel showed immunohistochemistry staining of midkine in lung tissue in WT, AAV.Veh and AAV.RNAi.MK group (*n* = 6 in each group). **b** Midkine and ACE protein levels in lung tissue determined by Western blots in control mouse (WT), mouse transduced with vehicle AAV (AAV.Veh) and mouse transduced with AAV carrying midkine RNAi sequence (AAV.RNAi.MK) (*n* = 6 in each group). **c** Immunohistochemistry staining of midkine in lung tissue of control mouse (WT) and mouse transduced with AAV midkine RNAi (AAV.RNAi.MK) with sham operation (upper panel), and the respective group mouse with CLP (lower panel) (*n* = 6 in each group). **d** H&E staining of control mouse (WT), mouse transduced with vehicle AAV (AAV.Veh) and mouse transduced with AAV carrying midkine RNAi sequence (AAV.RNAi.MK) in CLP vs. sham mouse were shown. Quantitative assessment by lung histopathological score was presented (*n* = 6). **e** BALF analysis from control mouse (WT), mouse transduced with vehicle AAV (AAV.Veh) and mouse transduced with AAV carrying midkine RNAi (AAV.RNAi.MK) in CLP vs sham mouse were shown (*n* = 6). Cell counts (upper left), TNF-α in BALF (upper right), protein contents in BALF (lower left) and lung wet/dry ratio (lower right) were shown (*n* = 6 in each group). **f** ACE hydrolytic activity determined by FAPGG in control mouse (WT), mouse transduced with vehicle AAV (AAV.Veh) and mouse transduced with AAV with midkine RNAi (AAV.RNAi.MK) in CLP vs. sham mouse was shown (left). ACE activity was measured at indicated time point. Angiotensin I was added to lung homogenization and the converted angiotensin II was determined (right) (*n* = 6 in each group)

Targeted abrogation of pulmonary midkine alleviated acute lung injury in CLP mouse. Histochemistry analysis indicated prominent pathophysiological injury in pulmonary tissue induced by CLP model (*n* = 6), including inflammatory cell infiltration, alveolar septa interstitial edema, congestion, hemorrhage and exudations (Fig. 3d).

Cell counts and TNF-α was significantly increased in BALF analysis (Fig. 3e), and pulmonary MPO activation was enhanced in CLP (*n* = 6) compared with sham (*n* = 6), implicated prominent neutrophil infiltration in CLP model. The lung wet/dry ratio of CLP mouse lung was increased, and protein contents in BALF was elevated, implying pulmonary edema and pulmonary vascular permeability increase.

We transduced AAV interfering midkine expression to mouse lung with trans-orotracheal instilment. The AAV transduction efficacy was validated by immunofluorescence staining (*n* = 6) (Fig. 3a), and midkine deletion efficacy was determined by Western blot (Fig. 3b) and immunohistochemistry (Fig. 3c).

Conditional deletion of midkine in pulmonary tissue attenuated lung injury, including reduced alveolar septa edema and intra-alveolar exudations in histochemistry analysis (Fig. 3d). The MPO activity in lung tissue was also decreased after midkine abrogation, suggesting alleviated neutrophil infiltration. The BALF analysis revealed that, by interfering midkine expression, cell counts and TNF-α in the BALF were significantly reduced (Fig. 3e), implicating resolved inflammation in lung tissue. Lung wet/dry ratio and protein contents in BALF was also decreased, indicating alleviated lung edema and pulmonary vascular permeability. All these results suggested that pulmonary midkine ablation could mitigate lung injury in sepsis.

### Midkine induced acute lung injury was mediated by ACE system in CLP mouse

Plasma ACE [ng/ml, 454.1 (378.1–500.3) vs. 53.37 (46.34–63.84), *P* = 0.0022] and angiotensin II [7.2 (6.8–9.6) vs. 1.9 (1.2–3.3), *P* = 0.002] were obviously increased in CLP group (Fig. 2b, c), midkine and ACE mRNA and protein level in lung tissue were markedly increased (Fig. 2d, e) in CLP mouse compared with sham. The hydrolytic ability of ACE was greatly enhanced in CLP (Fig. 3f). However, when pulmonary midkine expression was ablated, both ACE protein level in lung tissue and its hydrolytic activity was drastically decreased (Fig. 3b and f), implicated its regulatory roll on ACE system.

### Midkine induced ACE-Ang II pathway stimulation in vitro

**Fig. 4:**
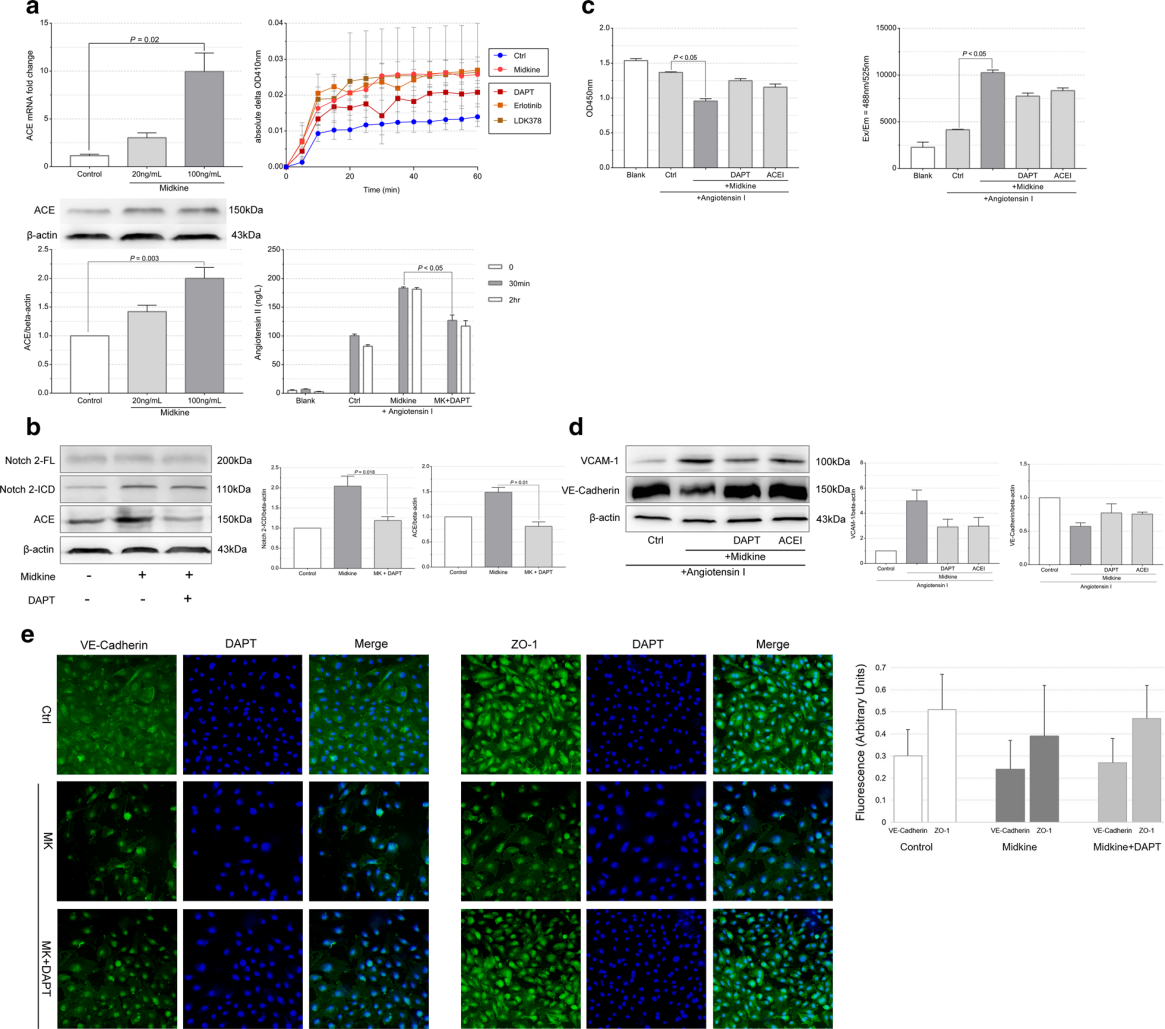
Pulmonary microvascular endothelial cell injury was mediated by ACE system via Notch 2 receptor. **a** ACE mRNA level in pulmonary microvascular endothelial cell (PMVEC) in control group, cells stimulated by midkine (20 ng/mL and 100 ng/mL), and normalized to β-actin (upper left). ACE protein level in PMVEC lysates in control group, cells stimulated by midkine (20 ng/mL and 100 ng/mL). Quantitative analysis using densitometry normalized to β-actin was shown (lower left). PMVEC ACE activity determined by FAPGG in control group, cells stimulated by midkine, and cell stimulated by midkine and blocked with inhibitors targeting Notch 2 (DAPT), ALK (LDK378) and EGFR (Erlotinib), respectively. ACE activity was measured at indicated time point (upper right). Angiotensin II in the supernatant was determined after angiotensin I was added to the culture medium, in control group, cells stimulated with midkine, and cells stimulated with midkine blocked by DAPT (lower right). **b** Notch 2 and ACE protein level in PMVEC in control group, cells stimulated with midkine (100 ng/mL), and cells stimulated by midkine and blocked by DAPT. Quantitative analysis of full length (FL) and intra-cellular domain (NICD) of Notch 2 and ACE, using densitometry, normalized to β-actin. **c** Cell viability determined by CCK-8 assay of PMVEC in blank, control group, cells stimulated by midkine, cells stimulated by midkine and blocked by DAPT and ACEI respectively. Reactive oxygen species (ROS) production in PMVEC in blank, control group, cells stimulated by midkine, cells stimulated by midkine and blocked by DAPT and ACEI respectively. **d** The expression of inter-cellular adhesive molecules including VCAM-1 and VE-cadherin were determined by Western blots. The expression of VCAM-1 and VE-cadherin in PMVEC were shown in control group, cells stimulated by midkine, cells stimulated by midkine and blocked by DAPT and ACEI, respectively. **e** Immunofluorescence staining of VE-Cadherin and ZO-1 in PMVEC in control group, cells stimulated by midkine and cells stimulated by midkine but prohibited by DAPT

To further investigate the impact of midkine on ACE system in vitro, pulmonary microvascular endothelial cells were stimulated by midkine (100 ng/ml) for 24 h. The result showed that the ACE mRNA and protein level was significantly induced by midkine stimulation (Fig. 4a).

We then evaluated ACE hydrolytic activity, and the conversion of angiotensin II by after angiotensin I was added to culture medium. We found up-regulated ACE hydrolytic activity and the increased level of angiotensin II (Fig. 4a), which further consolidate the evidence that midkine activated ACE system.

### ACE activation induced endothelial injury was by midkine stimulation via Notch 2 receptor

We used small molecular inhibitors targeting candidate midkine receptors including ALK, EGFR and Notch 2, and the results showed that the ACE activity was suppressed when Notch 2 receptor was inhibited (Fig. 4a), and the expression of ACE protein was also decreased by midkine stimulation after Notch 2 block (Fig. 4b).

The viability of the endothelial cells was measured by cholecystokinin (CCK)-8 assay, and the results indicated that the midkine stimulated ACE—angiotensin II activation could decrease the viability of endothelial cells (Fig. 4c).

Vascular endothelial injury was characterized by increased expression of adhesive molecules and damaged inter-cellular junction by ROS injury induced by angiotensin II. We thus detected the ROS production upon midkine stimulation and the observed that ROS production was significantly increased after midkine stimulation and was reduced after Notch 2 inhibition. The expression of inter-cellular adhesive molecules including ICAM-1 and VCAM-1 were increased by midkine (Fig. 4d), implicating the abnormally activated endothelial cells. Also, immunofluorescence staining of VE-Cadherin and ZO-1 was detected, which both had a trend to decrease after Notch 2 inhibition (Fig. 4e).

All these results above collectively elucidated that Notch 2 receptor participated in the vascular endothelial injury mediated by ACE system stimulated by midkine.

## Discussion

In this study, we found that midkine was remarkedly elevated in sepsis, and was associated with increased expression of ACE and severity of lung injury both in patients and CLP models. Targeted abrogation of midkine expression in lung tissue could ameliorate lung injury in vivo*.* The mechanism might be the suppress of ACE activity via Notch 2 receptor and decreased angiotensin II release.

Midkine has been proved to be elevated both in plasma and tissues during sepsis [11, 12], however, its effects on the organ function was under debate, midkine seemed to have an organ-dependent effect, which showed a protective effect in acute cardiac infarction [28], with angiogenesis promotion [29] and vascular endothelial repairment [30], but a deleterious effect in kidney [31], lung injury [32, 33] and autoimmune diseases [34]. In the present study, we found high circulating midkine was correlated to the severity lung injury induced by sepsis. Elevated midkine expression in the lung was associated with more significant pulmonary pathological alterations and more severe inflammation. Targeted midkine inhibition in the lung could mitigate lung injury in CLP, implicated its detrimental role in the lung injury during sepsis. This result was consistent with our clinical findings, that septic patients complicated with moderate to severe ARDS had higher level of plasma midkine compared with those with none or mild ARDS patients [12]. Interestingly, organ-specific interfering of midkine did not significantly affect circulatory ACE, which may possibly partially contribute to the activation of local RAS system [35].

We further investigated the mechanism by which midkine mediated the lung injury and found that ACE system activation could contribute to this process, of which was associated with midkine expression. By interfering midkine expression in lung tissue with the transduction of the adeno-associated virus with RNA intervening midkine expression, an obvious decrease in ACE protein was found and its hydrolytic activity was suppressed, implying the modulatory effects of midkine on ACE. This mechanism has also been be demonstrated in a stretch induced mouse lung injury by Zhang et al., in which midkine induced the proliferation of fibrotic cell and promoted fibrosis in the sub-acute or late phase of acute lung injury [33], while in our study, we investigated the acute phase and effect of ACE and angiotensin II, through NADPH uncoupling and ROS generation, and found increased ROS in vascular endothelial cells challenged by midkine added with angiotensin I.

It was widely accepted that midkine, as a multi-functional cytokine, also has other effects apart from RAS stimulation, including promotion of neutrophil aggregation and chemotaxis [10]. In the present study, we detected MPO activity in the lung tissue, which reflected the neutrophil infiltration in the pulmonary interstitial tissues, and was also decreased in CLP mouse with pulmonary midkine inhibition, however, we still could not verify whether this preservation in lung injury was due to the resolved inflammation or the direct impact of midkine inhibition, which needed further investigation.

As we further investigated the mechanism by which midkine affected pulmonary function in vitro using pulmonary microvascular endothelial cells. Stimulated by midkine, and after angiotensin I was added to the culture medium, we found obvious impairment of the vascular endothelial cell layer integrity, with decreased VE-cadherin expression and up-regulated adherent molecule VCAM-1, which suggested exacerbated endothelial injury, activated inflammation and increased endothelial permeability [8]. We also found the activated ACE system and increased production of angiotensin II, and stimulated ROS activity, implicated the role ACE in this process.

Candidate receptors including ALK, EFGR and Notch 2 have been reported to be involved in the signal pathway transduction through which ACE was activated [20–22, 36]. In this study, we examined the receptors that was potentially related to the process of ACE activation induced by midkine and found that after the Notch 2 was suppressed, the protein expression and hydrolytic activity of ACE was significantly suppressed, suggesting its role in the ACE activation. Notch 2 had been reported to be enrolled in epithelium-mesothelium transition (EMT) [22] and chemoresistance of pancreatic carcinoma [37], and participated in the process the pulmonary fibrosis in ARDS [33]. Our study further demonstrated the role of Notch 2 in midkine mediated ACE system activation and subsequent endothelial injury.

Our study had several limitations. Although we saw no differences in the circulating midkine and ACE in plasma, we did not measure the blood pressure of the mice, considering that angiotensin II was a vessel constrictor. Secondly, we didn't measure any parameter with ALK, EFGR inhibitors other than ACE activity. Although these blockers were ineffective in reducing ACE activity, they could exert a possible protective effect by acting on other potential targets that could be activated by midkine. Thirdly, we investigated the impact of midkine in sepsis and its role in sepsis induced injury, and as we know, midkine was extensively distributed in both lung and kidney, since we only evaluated the impact of midkine in the lung, we could further explore the effect of midkine in the lung, and its relevant mechanism. in the auto-immune induced kidney injury [38], midkine was reported to be positively associated with the deteriorations of the kidney function and exacerbation of pathological changes [31], so we extrapolated that midkine was possibly involved in the process of kidney injury and subsequent kidney dysfunction, which needed further investigation.

## Conclusions

Pulmonary midkine inhibition ameliorates sepsis induced lung injury, which might via ACE/Ang II pathway and the participation of Notch 2 in the stimulation of ACE.

## Supplementary Information

**Additional file 1**: **Fig S1**. Flowchart. ICU, intensive care unit; SLE, systemic lupus erythematosus; SOFA, sequential organ failure assessment.

## Data Availability

All data generated or analysed during this study are included in this published article.

## Abbreviations

AAV: Adeno-associated virus
ACE: Angiotensin-converting enzyme
ALI: Acute lung injury
ALK: Anaplastic lymphoma kinase
BALF: Bronchial alveolar lavage fluid
BCA: Bicinchoninic acid
BSA: Bovine serum albumin
CLP: Cecal ligation and puncture
DMSO: Dimethyl sulfoxide
ECGS: Endothelial cell growth supplement
EGFR: Epithelial growth factor receptor
ELISA: Enzyme linked immunosorbent assay
EMT: Endothelial mesenchymal transition
FAPGG: N-[3-(2-furyl) acryloyl]-L-phenylalanyl-glycyl-glycine
FBS: Fetal bovine serum
GFP: Green fluorescence protein
HRP: Horseradish peroxidase
ICU: Intensive care unit
IQR: Interquartile range
LPS: Lipopolysaccharide
MK: Midkine
MODS: Multiple organ dysfunction syndrome
MPO: Myeloperoxidase
PBS: Phosphate buffer solution
PCR: Polymerase chain reaction
PVDF: Polyvinylidene fluoride
RAS: Renin angiotensin-converting enzyme system
ROS: Reactive oxygen species
SD: Standard deviation
SDS-PAGE: Sodium dodecyl sulfate—polyacrylamide gel electrophoresis
SE: Standard error
TNF-α: Tumor necrotic factor alpha
VCAM-1: Intercellular adhesive molecule
