# Supplementary figures and images for: Pulmonary midkine inhibition ameliorates sepsis induced lung injury
Source: J Transl Med. 2021 Feb 27;19:91. doi: 10.1186/s12967-021-02755-z (PMC7913048; doi:10.1186/s12967-021-02755-z)

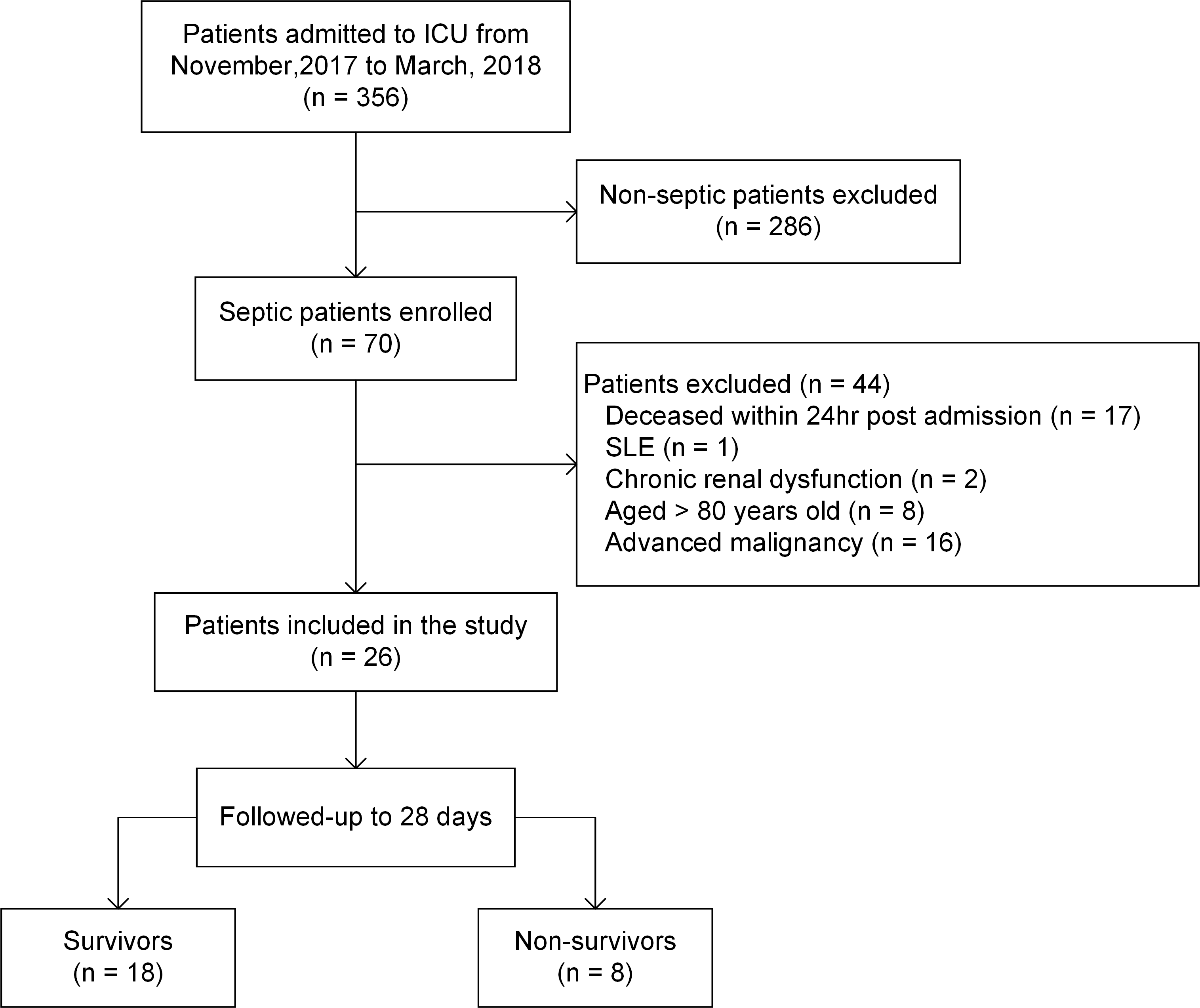

Supplement: Supplementary file 1 — Additional file 1: Fig S1. Flowchart. ICU, intensive care unit; SLE, systemic lupus erythematosus; SOFA, sequential organ failure assessment. [file 12967_2021_2755_MOESM1_ESM.tif]
